# Listen to the voices of nurses: the role of community chief nurses and registered nurses in the provision of care for older people in Sweden during the COVID-19 pandemic – a cross-sectional study

**DOI:** 10.1186/s12877-023-04652-0

**Published:** 2024-02-02

**Authors:** Anna Swall, Lena Marmstål Hammar, Anne-Marie Boström

**Affiliations:** 1 School of Health and Welfare, Dalarna University, Högskolan Dalarna, 791 88 Falun, Sweden; 2 School of Health, Care and Social Welfare, Mälardalen University, Box 833, 721 23 Eskilstuna/Västerås, Sweden; 3 Division of Nursing, Department of Neurobiology, Care Science and Society Karolinska Institute, Alfred Nobels Allé 23, 141 83 Stockholm, Huddinge Sweden; 4 Medical Unit Aging, Theme Inflammation and Aging, Karolinska University Hospital, Stockholm, Sweden; 5 R&D Unit, Stockholms Sjukhem, Stockholm, Sweden

**Keywords:** Community chief nurse, COVID-19, Nursing care, Medical care, Older people, Registered nurse, Pandemic

## Abstract

**Background:**

During the pandemic in Sweden, the aim was to protect older people, especially those among them who were sick, frail and vulnerable in residential care facilities. A ban was put on visits at all residential care facilities in March 2020 to prevent the spread of infection among the older people. This study aims to describe the experiences of Community Chief Nurses and Registered Nurses who provided medical and nursing care for older people in residential care facilities and home care during the first wave of the COVID-19 pandemic, and to examine factors associated with the quality of care.

**Methods:**

The study has a mixed method cross-sectional design (STROBE). Data were collected using a web-based survey that comprised two questionnaires, for Community Chief Nurses and Registered Nurses developed for the study. Data were analysed using descriptive statistics and logistic regression models, as well as qualitative content analyses.

**Results:**

The majority of Community Chief Nurses reported adequate opportunities to work with management to handle the COVID-19 pandemic. The Registered Nurses reported that the quality of care, as well as the person’s safety, was negatively affected during the pandemic. Factors associated with good care were as follows: information-sharing; ability to comply with hygiene practices; competence in how to care for older persons with COVID-19; a physician at bedside assessing their health; and support from frontline managers.

**Conclusion:**

The study highlights crucial facets that care organizations must address to enhance their readiness for future pandemics or disasters, ensuring the security and well-being of the older people.

**Supplementary Information:**

The online version contains supplementary material available at 10.1186/s12877-023-04652-0.

## Background

On March 11, 2020, World Health Organization (WHO) declared COVID-19 to be a pandemic [1]. Compared with the younger population, older people (70 years and older) [2] were shown to be affected more severely by the virus since they experienced higher levels of mortality and more complications [2, 3] Restrictions were implemented globally to curb the spread of the virus and safeguard vulnerable populations [4].

Sweden has 290 municipalities, and each is responsible for the healthcare and social care of older people in its residential care facilities (RFC) and home care [5]. In 2018, around 82,000 individuals aged 65 and older resided in an RCF, while approximately 144,000 received home care [6]. Most of these individuals are vulnerable and have extensive care needs [6, 7]. Since RCFs provide both healthcare and social care, they are regulated under two acts; the Health Care Act [8] and the Social Services Act [9]. Nationally in RCFs and home care, there are about 105,000 assistant nurses (with basic education in care for older people at the upper-secondary school level), about 60,000 care aides (no adequate formal education) and about 16,000 registered nurses (RNs). A community chief nurse (CCN) has the highest responsibility for the provision of medical and healthcare in the municipality and is responsible for ensuring that good and safe care is provided within the responsibilities for the municipality [10]. RN responsibilities include the everyday care of older people and clinical decisions relating to this. RNs work to improve, maintain and/or help people regain their health and achieve the best possible state of well-being and quality of life until their time of death [10]. RNs plan, provide and follow up on care systematically so that they can provide the best possible support in complex care situations [11].

A Swedish report [12] examining RNs caring for older people during the pandemic reported an increased workload due to staff shortages, inadequate teamwork and communication with staff and management, insufficient support, and heightened work-related stress, which impacted their ability to recover and consequently affected their personal lives. Other research on nursing staff in general (including RNs, assistant nurses, and care aides) caring for older people has also indicated increased stress, anxiety, depression, and post-traumatic stress syndromes [13–16]. These issues have been correlated with poor working conditions and a lack of skills and knowledge related to COVID-19 [17, 18]. Additionally, difficulties in adhering to guidelines to prevent virus spread were identified, as well as rapidly changing guidelines that created challenges in translating them into practice and ensuring staff education [19].

During the first wave of the COVID pandemic, Sweden stood out among the Nordic countries (Denmark, Finland, Norway and Sweden) since it had an infection rate that was three times higher than the others had despite an equal rate of municipal infection [20].

At the beginning of 2020, about 7,000 people in Sweden had died as a result of COVID-19: of these, about 90% were older, with 50% living in an RCF [21]. To protect older people, especially among them who were sick, frail and vulnerable in RCFs [21, 22], a ban was put on visits at all RCFs in March 2020 to prevent the spread of infection among RCF residents [19]. How these restrictions functioned in terms of preventing the spread of the infection and supporting the staff to be able to perform good quality of care need to be investigated. During the pandemic, the voices of the RNs and the CCNs were absent (or were not listened to) despite the fact they were ultimately responsible for the care of older people [23]. This absent of the voices of RNs is also highlighted by Rasmussen et al. [24]. To get a better understanding and knowledge what actions that supported or hindered the staff in their work to stop the spread of the infection during the COVID-19 pandemic from the RNs’ perspective are of great importance for future health crisis in society, and particular in the care of older people. Therefore, the aim of this study was to describe the experiences of CCNs and RNs who provided medical and nursing care for older people in RCFs and home care during the first wave of the COVID-19 pandemic, and to examine factors associated with the quality of care.

## Method

### Design

The study has a mixed method cross-sectional design consisting of questionnaires with close- and open-ended questions targeting CCNs and RNs working in the care of older people.

The checklist of guidelines for cross-sectional design, STROBE (EQUATOR) research reporting checklists have been uploaded).

### Study setting and sample

Inclusion criteria for the participants were as follows: RNs or CCNs who had been working in the municipal care of older people during the first wave of the COVID-19 pandemic in Sweden in the spring of 2020, which included RCFs as well as home care.

### Data collection

The data collection involved the employment of two questionnaires, one developed for CCNs and one for RNs. They focused on these areas: *Prerequisites for the prevention of the spread of the virus; Support for RNs and staff*, *Prerequisites for the provision of good medical and nursing care* and *demographic data*. The questionnaire for the CCNs had five questions, of which four included an open-ended response. The questionnaire for the RNs had 16 questions, of which nine included an open-ended response. Three questions were the same in both questionnaires, and the remaining questions were more specific in terms of the roles and responsibilities the respondents had as CCNs and RNs: see the Appendix.

Questions pertaining to infection control and management of individuals infected with COVID-19 were derived from those used by the Swedish Public Health Authority. The remaining questions, developed by the authors specifically for this study, focused on the roles of CCNs and RNs as well as care provision during this period. Additionally, demographic data, including educational levels, years of experience as an RN, and time spent in their current role, were collected. RNs with PhD and involved in research of the care of older people, as well as and representatives from Swedish Society of Nursing (SSN) read the questions and response alternatives to check the readability and clearness of the items. Given the urgency of the pandemic, we deemed it necessary to conduct data collection promptly, precluding any additional testing of the formulated questions, no further testing of the developed questions was made.

The participants were recruited via SSN, which sent out an invitation and a link to the questionnaire to all their members to take part in the study in their monthly newsletter and in social media. The questionnaires were open for responses from the beginning of June 2020 until the end of August 2020. Respondents could access the link to the electronic questionnaires that was published on the SSN website.

### Dependent and independent variables

The two questions that relate to whether it was possible to provide good medical care and nursing care reported by the RNs were chosen as dependent variables for the regression analyses (Table 1). The independent variables were identified from the three categories as *Prerequisites for the prevention of the spread of the virus; Support for RNs and staff;* and *Prerequisites for the provision of good medical and nursing care* (Table 1).

### Data analysis

#### Statistical analysis

The data from the two questionnaires were analysed using SPSS version 25. Missing data on the questions with response alternatives varied between 1 and 2 respondents for the 208 CCNs and 1 and 17 respondents for the 909 RNs. Due to the small proportions of missing data in the two samples, no imputation was performed. The response alternatives were dichotomised into high versus low for the questions (Table 1).

Descriptive statistics were used to present mean, standard deviation (SD) and percentage. Bivariate analyses were conducted using chi-square and student’s t-test. A logistic regression model was used to examine the associations between the two dependent variables quality of medical care and quality of nursing care and the eight independent variables. First, univariate logistic regression models were conducted to examine the associations between the two dependent variables and the eight independent variables. Second, the independent variables that were significantly associated in the univariate logistic models with the dependent variable were included in the multiple logistic regression model for each dependent variable. A *p*-value <0.05 was considered as statistically significant.

**Table 1:** Dependent and independent variables

	Response alternatives	Categorisation of response alternatives
**Dependent variables**		
Opportunities to provide good medical care during the pandemic have been affected	Always To a high extent To a low extent Seldom/Never	High extent (always and to a high extent) Low extent (low extent and seldom/never)
Opportunities to provide good nursing care during the pandemic have been affected	Always To a high extent To a low extent Seldom/Never	High extent (always and to a high extent) Low extent (low extent and seldom/never)
**Independent variables**		
* Prerequisites for the prevention of the spread of the virus*		
Experienced problems getting information about COVID-19 to staff	Yes No	Yes No
Staff have problems following and applying basic hygiene practices	Always To a high extent To a low extent Seldom/Never	High extent (always and to a high extent) Low extent (low extent and seldom/never)
Residential care facilities have been able to isolate people with symptoms of COVID-19	Yes No	Yes No
* Support for RNs and staff*		
Support from frontline manager	Always To a high extent To a low extent Seldom/Never	High extent (always and to a high extent) Low extent (low extent and seldom/never)
Support from CCNs	Always To a high extent To a low extent Seldom/Never	High extent (always and to a high extent) Low extent (low extent and seldom/never)
Recurring occasions when ethical issues have been discussed	Yes No	Yes No
* Prerequisites for the provision of good medical and nursing care*		
Physician has been present for assessments/decisions when a person has been suspected of having symptoms of COVID-19	Always To a high extent To a low extent Seldom/Never	High extent (always and to a high extent) Low extent (low extent and seldom/never)
Staff have competence to care for people with COVID-19 at the residential care facility	Always To a high extent To a low extent Seldom/Never	High extent (always and to a high extent) Low extent (low extent and seldom/never)

#### Qualitative analysis

Open-ended questions for both RNs and CCNs were included in the areas *Prerequisites for the prevention of the spread of the virus,* and *Prerequisites for the provision of good medical and nursing care.* See Appendix. The analysis was inspired by Elo and Kyngäs [24], and the responses of CCNs and the RNs to the questionnaires were analysed separately as were the divisions in each questionnaire regarding the areas *Prerequisites for the prevention of the spread of the virus* and *Prerequisites for the provision of good medical and nursing care*. The text was read several times so that a sense could be gained as to its content and meaning units, which were meanings of sections related to the study’s aim. The meaning units were labelled with codes based on the content of the units, and the codes were compared to its differences and similarities, and sub-categories and categories were developed (Table 2). To present the variations between the descriptions of the CCNs and the RNs, the results section presents them together in line with the predetermined areas: *Prerequisites for the prevention of the spread of the virus* and *Prerequisites for the provision of good medical and nursing care.* For an example of the analysis process, see Table 3.

**Table 2:** Categories and sub-categories

**Categories**	**Sub-categories**
Prerequisites for the prevention of the spread of infection	Handling protective equipment and guidelines
	Lack of competence
	Difficult work environment
	Deficiencies in hygiene practices and use of protective equipment
Prerequisites for the provision of good medical care and nursing care	Physician present
	Medical expertise absent
	Unethical medical procedure
	Ethical frustration
	Low level of education and language problems

**Table 3:** Example of the qualitative analysis process

**Meaning units**	**Codes**
Most of the staff lack basic nursing education. The staff have difficulty understanding the information and the importance of taking part in information and training.	No education
Initially, the difficulties were getting protective equipment.	Lack of equipment
Nursing staff have no training in how to care for persons with COVID-19. The person often lie flat on their backs. The nursing staff don't know how to put someone into the side position or into the heart position.	Lack of medical knowledge
No physicians on the unit for correct assessments/measures.	Lack of physicians

### Ethics approval and consent to participate

According to Swedish law, studies that do not involve sensitive data, such as data relating to health, politics or sexual orientation, do not require permission from the Ethical Review Authority (Ethical Review Act 2003:460, https://www.onep.se/media/2348/the_ethical_review_act.pdf ). This study was conducted in accordance with the ethical standards as set in the Declaration of Helsinki and its later amendments [25]. The respondents were given information about the purpose of the study and were told that participation was voluntary and that their answers were anonymous. By filling in the questionnaire, the informant gave their informed consent to participate in the study. They were also told that the results would be presented in a way that would prevent the identification of the respondents.

## Results

### Description of samples

The questionnaires were answered by 208 CCNs and 925 RNs. The CCNs had on average 25 years of experience as RNs, and an average of six years as CCNs. Just over two thirds were specialised nurses at the master level whose main focus areas were the care of older people, primary healthcare and psychiatric care.

Of the 925 RNs, 14 did not work in the care of older people; for this reason, they were excluded, which left 909 RNs who met the inclusion criteria. On average, these RNs had worked 10 years in RCFs or in home healthcare, and 319 (35%) had specialized nursing training at the level of master; of those, 117 (13%) were specialised in the care of older people and 113 (10%) in psychiatric care, midwifery, anaesthesia or pre-medicine.

### Prerequisites for the prevention of the spread of infection

#### The experiences of CCNs

All CCNs described good conditions for preventing the spread of COVID-19: for example, good collaboration at the municipal-level of management, as well as in managerial groups and with the healthcare organisation in the region, and also the Swedish authority that works with infection control, Infection control unit. Nearly all CCNs (98%) reported that specific guidelines on preventing the spread of COVID-19 had been developed and were disseminated within the organisation. But the implementation of the guidelines and information to staff for preventing the spread of the virus to frontline staff varied between municipalities. In some units and municipalities, the implementation was successful, but in about 2/3 units it failed. 

In the open-ended questions, some CCNs described feeling that access to protective equipment was good; that the introduction and implementation of basic hygiene practices for staff had worked well; and that there had been good collaboration with primary healthcare and the physician. However, they highlighted a lack of healthcare competence among managers in RCFs and home care settings. The majority of the CCNs appreciated that there were staff members in the organisation who had the necessary competence to care for people with COVID-19, but there were too few. Added to this, some CCNs raised the issue of the level of training of some care staff. Some had no training at all, which in turn meant that practices and guidelines were not fully followed or applied.

*“What is missing is nursing and medical care competence in top management. The social director lacks nursing and medical care competence. New decisions must often be made when the CCN becomes aware of these.” (CCN)*

Other CCNs described how their work became more difficult, which affected its quality and the ability to get information out early about correct basic hygiene practices. CCNs described how RNs had more work responsibilities than they were able to manage: they had more older people to care for and were also in charge of handling infection-control equipment and supervising staff in basic hygiene practices.

*“Practices for how and when to use protective equipment. Instructional videos and web-training about COVID-19 came early on in the pandemic, but many have still had difficulty understanding instructions due to language problems and a low level of education.” (CCN)*

The ambition to provide the staff, the units and the whole organisation with directives and, for example, instructional videos was good. However, in some situations, this failed as a result of other factors, which was described by the CCNs as frustrating. The CCNs gave examples of these factors by pointing out the fact that many RNs can be responsible for 400-600 older people during an evening, night or weekend work shift. This jeopardised the person’s safety in situations where staff were already working under pressure.

#### The experiences of RNs

Nearly all RNs (96-99%) reported that the organisation in which they worked had access to gloves, plastic aprons, hand disinfectant and surface disinfectant at the beginning of the first wave. A lower proportion of RNs reported having access to facemasks (71%), visors (55%) and goggles (34%). In the later stages of the first wave, all RNs reported that they had access to the necessary protective equipment.

Forty percent of the RNs experienced problems sharing information with staff about COVID-19 at the unit level (Table 4). Thirty-six percent of the RNs also reported that staff always or to a large extent had difficulties following and applying basic hygiene practices mainly as a result of their lack of knowledge. The open-ended question showed that staff with low competence had difficulty understanding information about isolation, hygiene practices and barrier care.

*Problems with a lack of knowledge among staff. Older people who we cannot/must not lock in/leave in their apartments to go to other people even though they have symptoms.” (RN)*

In the open-ended questions, some RNs described how they were convinced that the infection spread through staff in the units because of their lack of competence when it came to the application of hygiene practices, the use of protective equipment and the fact that the same staff worked in different units and possibly spread the virus to older residents. RNs also described how staff sometimes worked despite they themselves having symptoms. In addition, there were language issues since some staff had a low level of Swedish language skills and did not fully understand the information. This is described as affecting the quality of care and the person’s safety in light of the spread of infection.

Overall, there was a lack of staff during this period; however, RNs described how they were commonly able to find solutions so that they could manage. They also described adhering to the restrictions, restricting visits and cooperating with other RNs to address such issues as isolation. The majority (87%) of RNs reported that they were able to isolate people with COVID-19 symptoms. However, they also described how their RCF was not designed to deal with this situation although they still tried to isolate those who had been infected.

### Support for RNs and staff

About half of the RNs reported receiving support in their role during the pandemic from a CCN (22% always and 35% to a high extent) (Table 4). Two thirds of the RNs (28% always and 43% to a high extent) reported receiving support from their frontline manager. About two thirds of RNs reported that their manager was an RN. The remaining RNs reported that their frontline managers were professionals such as sociologists, occupational therapists, physiotherapists and assistant nurses. About 15% of RNs did not report the profession of their manager. The RNs who reported that their frontline manager was an RN also reported receiving support from their manager to a higher extent than those whose manager was not an RN by profession (76% compared with 63%; *p*<0.001).

More than half (58%) of the RNs reported that during the pandemic, recurring meetings were held to discuss the ethical issues of COVID-19. Meetings for reflection between RNs and staff were arranged as well as for RNs solely. These focused on processing the situation and also on exchanging experiences and improving the care in their units.

**Table 4:** Descriptions of the prerequisites for the prevention of the spread of infection, for the provision of good medical care and nursing care, and for support for registered nurses and staff, and comparisons between independent variables and opportunities to provide good medical and nursing care or not, as reported by registered nurses.

	Total Sample *N*=909	Opportunities to provide good medical care during the pandemic have been affected. Answer: Always and to a high extent. *n*=419 n (%)	Opportunities to provide good medical care during the pandemic have been affected. Answer: to a low extent and seldom. *n*=482 n (%)	*P*-value	Opportunities to provide good nursing care during the pandemic have been affected. Always and to a high extent. *n*=385 n (%)	Opportunities to provide good nursing care during the pandemic have been affected. Answer: To a low extent and seldom. *n*=514 n (%)	*P*-value
**Prerequisites for the prevention of the spread of infection**							
Have you **experienced problems** getting information out to staff about COVID-19? Answer: yes	361 (40%)	195 (46.7%)	162 (33.8%)	<0.001	199 (51.7%)	158 (30.7%)	<0.001
Do you **find it difficult for staff** to follow and apply basic hygiene practices? Answer: Always and to a high extent	323 (36%)	193 (46.1%)	130 (27.0%)	<0.001	198 (51.2%)	125 (24.2%)	<0.001
Has the residential facility been able to **isolate people with symptoms** of COVID-19? Answer: Always and to a high extent	788 (87%)	344 (82.3%)	440 (91.7%)	<0.001	313 (81.1%)	474 (92.2%)	<0.001
**Support to RNs and staff**							
Support from frontline manager Answer: Always and to a high extent	637 (70.8%)	261 (62.3%)	376 (78.2%)	<0.001	244 (63.0%)	395 (76.7%)	<0.001
Support from Community Chief Nurse Answer: Always and to a high extent	510 (56.8%)	202 (48.3%)	308 (64.2%)	<0.001	187 (46.6%)	324 (63.0%)	<0.001
There have been recurring occasions when ethical issues have been discussed in the organisation. Answer: Yes	521 (58%)	236 (56.6%)	283 (59.1%)	0.452	217 (56.2%)	303 (59.2%)	0.373
**Prerequisites for the provision of good medical care and nursing care**							
Physicians have been present for assessments/decisions concerning the older person with suspected/confirmed COVID-19? Answer: Always and to a high extent	306 (34%)	112 (27%)	193 (41.1%)	<0.001	105 (27.5%)	200 (39.7%)	<0.001
Competence to care for people with COVID-19 at the residential facility. Answer: Always and to a high extent	642 (72%)	25360.7%)	387 (81%)	<0.001	223 (57.9%)	419 (81.8%)	<0.001

### Prerequisites for the provision of good medical care and nursing care

#### The experiences of CCNs

Of the CCNs, 25% reported that the quality of medical care was negatively affected during the pandemic, and 22% reported that the quality of nursing care was affected. In the open-ended questions, the CCNs described how collaboration with the physician improved during the first wave. However, there were descriptions that visits from the physician were completely cancelled at some units and that RNs were left to make decisions on medical issues without being able to contact a physician. In some cases, the CCNs described how the physician prescribed palliative care over the phone to older people they had not met. CCNs also described how even greater demands were made of RNs during the pandemic in situations that were already stressful. This involved providing guidance to staff in, for example, hygiene practices and isolation care as unit staff were not sufficiently competent in these areas.

#### The experiences of RNs

Nearly half (46%) of the RNs reported that the quality of medical care was affected during the pandemic (Table 4). A slightly lower proportion (42%) of the RNs felt that nursing care was negatively affected during the pandemic. One third answered that the physician was always (10%) or to a high extent (24%) present for assessments and decisions concerning older people with suspected or confirmed COVID-19. One third (33%) answered that the physician was seldom/never present.

In the open-ended questions, the RNs described how physicians had not been at their unit for, in some cases, up to three months and that all contact had been by phone or video call. RNs like CCNs described how the physician sometimes prescribed “palliative prescriptions” as a standard practice to the older people infected with COVID-19 even though they had not been in a palliative stage prior to the COVID-19 infection. In these situations, the RNs described a sense of being alone in the responsibility they were left with when they argued that the older people in their care should not be in palliative care. They also described feeling frustrated about not being able to provide the older person in their care with, for example, oxygen as this was not available at most RCFs. The situations were described in terms of abandonment and of being alone to make difficult decisions when the older people at the RCFs died.

*“I have worked with death all my life, both with children, young and old. But this was the worst imaginable scenario.” (RN)*

In addition, the RNs described how the physician in some cases prescribed “no CPR” for the older people with COVID-19 without having informed either the person or their relatives. RNs described severe ethical struggles in their attempts to advocate for the rights of the older people to receive treatment while also having to deal with their own thoughts and fears.

### Factors associated with the ability to provide good medical care and nursing care

The univariate logistic regression models revealed that all independent variables were significantly associated with the two dependent variables except for the independent variable “Recurring occasions when ethical issues have been discussed” (Table 5).

In the multiple logistic regression model with the dependent variable “Ability to provide good medical care”, these four independent variables were significantly associated with the dependent variable: “Competence to care for people with COVID-19” (OR=1.92); “Support from frontline manager” (OR=1.69); “Staff’s ability to comply with hygiene practices” (OR=1.64); and “Physician at bedside assessing people” (OR=1.57).

In the second logistic multiple regression model, these four independent variables were significantly associated with the ability to provide good quality nursing care: “Staff’s ability to comply with hygiene practices” (OR=2.31); “Competence to care for people with COVID-19” (OR=2.09); “Information-sharing with staff” (OR=1.45); and “Physician at bedside assessing people” (OR=1.39).

The two independent variables “Ability to isolate people showing symptoms of COVID-19” and “Support from CCN” were significantly associated with the two dependent variables in the univariate analyses but not in the multiple logistic regression models.

**Table 5:** Logistic regression models regarding possibilities to provide good quality medical and nursing care during the pandemic.

	Possibilities to provide good quality medical care during during the pandemic Always and to high extent				Possibilities to provide good quality nursing care during during the pandemic Always and to high extent			
	Univariate logistic regression		Multiple logistic regression		Univariate logistic regression		Multiple logistic regression	
	OR	95% CI	OR	95% CI	OR	95% CI	OR	95% CI
Experienced problems reaching out with information about COVID-19 to staff (Yes vs No)	**1.72**	**1.31-2.25**	1.06	0.78-1.45	**2.41**	**1.83-3.17**	**1.45**	**1.06-1.98**
Difficult for staff to follow and apply basic hygiene routines (High extent vs Low extent)	**2.31**	**1.75-3.05**	**1.64**	**1.20-2.24**	**3.28**	**2.47-4.35**	**2.31**	**1.69-3.16**
Isolate persons with COVID-19 symptoms (Low extent vs High extent)	**2.37**	**1.57-3.56**	1.31	0.84-2.06	**2.76**	**1.83-4.17**	1.46	0.92-2.30
Support from front-line manager (Low extent vs High extent)	**2.17**	**1.62-2.91**	**1.60**	**1.15-2.22**	**1.93**	**1.44-2.58**	0.79	0.83-1.10
Support from Community Chief Nurse (Low extent vs High extent)	**1.92**	**1.46-2.50**	1.27	0.94-1.73	**1.81**	**1.38-2.36**	1.13	0.83-1.55
Recurring occasions when ethical issues have been discussed (No vs Yes)	1.11	0.85-1.44			1.13	0.86-1.48		
Physician has been presented to assess patients with symptoms of COVID-19 (Low extent vs High Extent)	**1.88**	**1.42-2.50**	**1.57**	**1.16-2.12**	**1.74**	**1.30-2.31**	**1.39**	**1.02-1.90**
Competence to care for persons with COVID-19 at the residential care home (Low extent vs High extent)	**2.76**	**2.04-3.73**	**1.92**	**1.37-2.69**	**3.27**	**2.42-4.43**	**2.09**	**1.49-2.93**

*OR* Odds Ratio, *CI* Confidence Interval; Statistically significant (*P*<0.05) indicated in bol

## Discussion

The aim of this study was both to describe the experiences of CCNs and RNs who provided medical and nursing care for older people in RCFs and in home care during the first wave of the COVID-19 pandemic, and to examine factors associated with quality of care. In general, the CCNs and RNs reported similar concerns regarding the effect on the quality of care for older people although the RNs reported to a higher extent problems with lack of knowledge and collaboration with staff and physicians than did the CCNs. The results from the logistic regressions point to three important aspects that will be discussed: perspectives on staff; collaboration with physicians; and support from frontline managers.

### Perspectives on staff

Most CCNs reported that guidelines to prevent the spread of the infection as well as to care for older people with COVID-19 worked well, although some CCNs pointed out problems implementing these guidelines in RCFs and home care. Similar results were found, stating that a lack of guidelines and teamwork regarding hygiene practices resulted in staff developing their own strategies [26]. To a greater extent than the CCNs, the RNs reported that the quality of care, as well as the person’s safety, were negatively affected during the pandemic. RNs reported a greater negative impact on care quality and patient safety during the pandemic compared to CCNs. Johansson-Pajala et al. [27] described heightened anxiety and loneliness among older people in RCFs during the first wave. Additionally, studies from other countries revealed similar findings, such as diminished person-centered care and reduced promotion of dignity for older people [28]. Schrack et al. [29] reported decreased social contact for persons with dementia in RCFs during the pandemic. In addition, Sweeney et al. [30] found similar results, which suggests that the care of older people was affected regardless of whether or not they had COVID-19. The older people who did not have the virus were neglected as a result of staff working under pressure. During the pandemic, failures in care were highlighted in Swedish media, which brought to light the issues in Sweden’s care of older people in the municipalities: some of these issues were inadequate staffing, non-continuity of staff and inadequate competence where it was needed. There was also an issue with the coordination of healthcare regional [4, 21]. Previous research, as well as our results, correspond with the report by the Swedish Corona Commission [21] that showed that the care of older people was majorly affected. Staff lacked the necessary training in how to care for older people, and there were major failures as a result of inadequate care brought about by COVID-19 in relation to such aspects as hygiene practices and end-of-life care.

Previous studies have indicated that a considerable number of care staff working with older people do not have Swedish as their first language [21, 30]. Our results highlighted the consequences of this situation, wherein a considerable portion of staff in RCF have Swedish as their second language. This can lead to communication issues and hinder their full comprehension of guidelines, restrictions, and new practices. This came to light in the corona commission report as well, and the Swedish government has now decided to place resources (about 30 million SEK) on the language (Swedish) training of staff [31]. One third of the RNs reported that staff had difficulties implementing basic hygiene practices. Hanna et al. [19] also found this issue in their study: where the roles of staff rapidly changed and came to include duties that they were not prepared for as they worked to prevent the spread of the virus. Both Sweeney et al. [30] and Hanna et al. [19] found that staff were afraid of spreading the virus to the older people in their care, to their families and to themselves as well, which corresponds to the responses to the open-ended questions in our study.

### Collaboration with physicians

Our findings revealed that 33% of the RNs reported that a physician was seldom or never present for bedside assessments or treatments. This issue was also highlighted in the report by the Corona Commission [21], which identified a substantial problem stemming from the fact that physicians are employed at the regional level, rather than the municipal or private level. This arrangement proved to be problematic for the organization of care. In our results, the RNs expressed a sense of loneliness in their struggle to provide high quality care for the older persons in their care. Bergqvist et al. [26] shed further light on this from the point of view of staff (nurse assistents), who described their sense of loneliness and of being disregarded as they were often left alone and did not receive adequate support from their managers, RNs and municipality-level management. In addition to this, the report of the Corona Commission [21] pointed out that inadequate staffing and competence resulted in an untenable work situation for both staff and RNs, which negatively affected the care and safety of older people in RCFs and home care during the pandemic. Our study, as well as that by Bergqvist et al. [26], describes a lack of appropriate nursing competence during the pandemic; however, the fact is that even not in the face of a pandemic, there are too few RNs working in the care of older people in Sweden before the pandemic. Only 9% of those employed in the care of older people in Sweden are RNs and as our results demonstrate, they may have responsibility for caring for between 400 and 600 older people during a work shift [32]. This means that it is impossible for them to work bedside, which became even more obvious during the pandemic when the older people needed advanced care.

### Support from frontline managers

About one third of the RNs described a lack of support from their managers; in contrast, those whose managers were RNs were to a greater extent satisfied with the support they received. Dissatisfaction with leadership and management has been confirmed by previous national and international research [33–38]. In Sweden, this may not be surprising, as frontline managers in the care of older people oversee a large group of staff (averaging 42 staff members in home care and 50 staff members in RCFs). Consequently, providing support to each staff member presents a considerable challenge [39]. The report of the Corona Commission describes a need for more advanced care and thus higher nursing and medical competences: these aspects were made apparent during the pandemic. However, this is nothing new since studies over the recent decade have shown this to be the case and have also raised the issue of inadequate staffing. Together, these severely affect the quality of care for older people. The report by The Health and Social Care Inspectorate [31] outlined how the lack of trained RNs and insufficient access to physicians meant that those with little training were left to perform work tasks that they did not have the professional skills to perform, and these staff members often also had poor Swedish language skills. IVO [31] states to be alarming since this greatly jeopardises the safety of older people. In other words, what our study and several other studies on the pandemic have found is that this issue exists regardless of whether there is a pandemic.

### Strengths and limitations

In total, 208 CCNs responded to the survey. In Sweden, there are approximately 400 CCNs; as such, the response rate was around 50%. The number of RNs who responded to the survey was 909, and according to the National Board of Health and Welfare, there were 13,983 RNs employed in the care of older people in Sweden in 2019. This means then that a limitation to this study could be the response rate among the RNs, which was approximately 6% and thus very low considering the high number of potential respondents. We did not ask the respondents about the place at which or the municipality in which they worked for reasons of confidentiality which meant that we could not combine and compare the responses of the CCNs and RNs by municipality.

Another limitation is that the survey was not tested among CCNs and RNs and evaluated prior to the data collection. Some of the questions in the survey were questions asked by the Public Health Authority in spring 2020. However, the number of responses to the open questions was high. Of RNs 93% and of CCNs 99% responded to the question about *Prerequisites for the prevention of the spread of infection*. Of the RNs and CCNs, 92% and 95% respectively responded to the question about *Prerequisites for the provision of good medical care and nursing care,* which gave more descriptive and substantial data about the situation for the two groups during the pandemic and also confirms the responses to the questions with fixed-response alternatives.

The answers of the CCNs to the open-ended questions were generally more positive in terms of the descriptions they gave of the care that was provided compared with the descriptions provided by the RNs. However, this may reflect the reality they worked in during the pandemic. There was a heavy burden on RNs due to the spread of the virus at RCFs and the fact they worked frontline. The role of the CCN was more administrative for example, they presented guidelines and instructions, and facilitated collaboration between the care organisations in the municipality and the region. The open-ended answers also give an idea of the experiences of CCNs and RNs in the first wave. Another limitation may be that the answers were not in-depth as no interviews were conducted, which can affect results. However, many respondents wrote answers on the open-ended questions that were very long and describing. The analysis of the open-ended questions was done in collaboration with all authors with the first author (AS) taking the lead. The analysis was discussed in the research team until consensus was reached so as to ensure trustworthiness.

## Conclusion and implications

The pandemic revealed a number of failures within Swedish healthcare relating to the care of older people: for example, a lack of competence and training, and insufficient medical expertise and resources. All these need to be addressed in policy and by decision-makers.

The pandemic underscored the urgent need for enhanced nursing and medical expertise, improved working conditions, updated regulations, and increased staffing in RCF. Our findings highlight the shortcomings that require attention. Research, education, and development are crucial for ensuring high-quality care for older people within municipalities, as well as for better preparedness in the face of upcoming health crises and climate changes.

### Supplementary Information

**Additional file 1. **Appendix.

**Additional file 2.** 

## Data Availability

The data are not publicly available due to restrictions in accordance with the Swedish Public Access to Information and Secrecy Act (2009:400). Requests for access to the dataset should be sent to the corresponding author and will be considered by the University’s data protection officer.

## Abbreviations

CCN: Community chief nurse
RN: Registered nurse
RFC: Residential care facilities
SSN: Swedish Society of Nursing
