# Supplementary material for: Listen to the voices of nurses: the role of community chief nurses and registered nurses in the provision of care for older people in Sweden during the COVID-19 pandemic – a cross-sectional study
Source: BMC Geriatr. 2024 Feb 2;24:127. doi: 10.1186/s12877-023-04652-0 (PMC10835959; doi:10.1186/s12877-023-04652-0)
Supplement: Supplementary file 1 — Additional file 1. Appendix. [file 12877_2023_4652_MOESM1_ESM.docx]

Appendix

| **CCN questionnaire** | **RN questionnaire** | **Response alternative** |
| --- | --- | --- |
| ***Prerequisites for the prevention of the spread of the virus*** | |  |
| Would you say that the units (residential care facilitates and home healthcare) in your organisation have the prerequisites for the prevention of the spread of Covid-19? | X | Open-ended |
| Has your organisation **given specific directions** to the units (residential care facilities and home healthcare) on how to prevent the spread of Covid-19? | X | Always  To a high extent  To a low extent  Seldom/Never |
| X | Did your organisation have the following protection equipment at the beginning of the pandemic?  Plastic aprons  Gloves  Visors  Goggles  Mouth guards  Hand disinfectant  Surface disinfectant | Yes/No |
| X | Did your organisation have the following protection equipment later on in the pandemic?  Plastic aprons  Gloves  Visors  Goggles  Mouth guards  Hand disinfectant  Surface disinfectant | Yes/No |
| X | Have you experienced problems getting **information** about Covid-19 out to staff? | Yes/No  Open-ended: If yes, please describe how |
| X | Would you say that staff have problems **applying basic hygiene practices**? | Always  To a high extent  To a low extent  Seldom/Never  Open-ended: If yes, please describe how |
| X | Has your organisation been able  **to isolate people who have Covid-19 symptoms**? | Yes/No |
| X | What actions has your organisation taken regarding Covid-19? | Open-ended |
| X | What would you say is the largest risk with the spread of Covid-19? | Open-ended |
| X | If you have had older people who have been diagnosed with Covid-19 in your organisation, how do you think the virus was first brought into your organisation? | Open-ended |
| ***Support for RNs and staff*** | |  |
| X | To what extent would you say that you have received support from your first-line manager during the pandemic? | Always  To a high extent  To a low extent  Seldom/Never |
| X | What profession does your first in-line manager have? | Open-ended |
| X | To what extent would you say that you received support from a Medical Registered Nurse in your municipality during the pandemic? | Always  To a high extent  To a low extent  Seldom/Never |
| X | Have there been recurrent opportunities to discuss ethical matters relating to Covid-19 in your organisation? | Yes/No |
| ***Prerequisites for the provision of good medical and nursing care*** | |  |
| Would you say that the provision of good medical care has been negatively affected during the pandemic? | Would you say that the provision of good medical care has been affected during the pandemic? | Always  To a high extent  To a low extent  Seldom/Never  Open-ended: If yes, please describe how |
| Would you say that the ability to provide good nursing care been affected during the pandemic? | Would you say that the provision of good nursing care has been affected during the pandemic? | Always  To a high extent  To a low extent  Seldom/Never  Open-ended: If yes, please describe how |
| Would you say that your organisation has the necessary competence to care for older people with Covid-19? | Would you say that your organisation has the necessary competence to care for older people with Covid-19? | Always  To a high extent  To a low extent  Seldom/Never  Open-ended: If no, please describe how |
| X | When an older person was suspected of having Covid-19, did a physician do a physical visit to examine and give a diagnosis? | Always  To a high extent  To a low extent  Seldom/Never |
